# The clinical consequences of a pre-hospital diagnosis of stroke by the emergency medical service system. A pilot study

**DOI:** 10.1186/1757-7241-20-48

**Published:** 2012-07-10

**Authors:** Ingela Wennman, Paula Klittermark, Johan Herlitz, Bodil Lernfelt, Mats Kihlgren, Claes Gustafsson, Per-Olof Hansson

**Affiliations:** 1 Department of Ambulance and Pre-hospital Emergency Care, Sahlgrenska University Hospital, Gothenburg, Sweden; 2 Department of Internal Medicine, Sahlgrenska University Hospital/Östra, Gothenburg, Sweden; 3 The Centre of Pre-hospital Research,Western Sweden, University College of Borås and Sahlgrenska University Hospital, Gothenburg, Sweden

## Abstract

**Background:**

There is still a considerable delay between the onset of symptoms and arrival at a stroke unit for most patients with acute stroke.

The aim of the study was to describe the feasibility of a pre-hospital diagnosis of stroke by an emergency medical service (EMS) nurse in terms of diagnostic accuracy and delay from dialing 112 until arrival at a stroke unit.

**Methods:**

Between September 2008 and November 2009, a subset of patients with presumed acute stroke in the pre-hospital setting were admitted by EMS staff directly to a stroke unit, bypassing the emergency department. A control group, matched for a number of background variables, was created.

**Results:**

In all, there were 53 patients in the direct admission group, and 49 patients in the control group. The median delay from calling for an ambulance until arrival at a stroke unit was 54 minutes in the direct admission group and 289 minutes in the control group (p < 0.0001).

In a comparison between the direct admission group and the control group, a final diagnosis of stroke, transient ischemic attack (TIA) or the sequelae of prior stroke was found in 85% versus 90% (NS). Among stroke patients who lived at home prior to the event, the percentage of patients that were living at home after 3 months was 71% and 62% respectively (NS).

**Conclusions:**

In a pilot study, the concept of a pre-hospital diagnosis of stroke by an EMS nurse was associated with relatively high diagnostic accuracy in terms of stroke-related diagnoses and a short delay to arrival at a stroke unit. These data need to be confirmed in larger studies, with a concomitant evaluation of the clinical consequences and, if possible, the level of patient satisfaction as well.

## Background

Acute ischemic stroke and acute myocardial infarction are severe consequences of cardiovascular disease. In both conditions, early treatment and early rehabilitation appear to improve outcome [1-4].

The introduction of thrombolysis in stroke treatment has considerably improved the acute care of stroke patients [5]. However, the vast majority of stroke patients are not eligible for thrombolysis or thrombectomy, often as a result of patient delay [6,7], and, in some hospitals, these patients are not given high priority at the emergency department (ED), thereby creating unnecessary delay before they reach the stroke unit and rehabilitation can be started.

One way to approach this dilemma is to introduce the concept of pre-hospital diagnosis, thereby bypassing the ED and transporting the patient directly to a ward with health care providers specifically educated for the treatment of the actual disease.

The experience of such a procedure is impressive with regard to acute myocardial infarction [8,9], but it is limited with regard to acute ischemic stroke.

Two major criteria are required for a procedure of this kind.

1. The patient is transported by the emergency medical service (EMS) and

2. The EMS health care providers have the capability to recognize stroke.

In the last decade, an increasing number of countries have equipped their EMS system with nurses or physicians in order to improve the accuracy of diagnostics and therapy [10]. In the city of Gothenburg, Sweden, each ambulance has at least one nurse on duty at all times, day and night.

The aim of this pilot study was to evaluate the feasibility of an ambulance nurse correctly diagnosing an acute stroke patient and, if so, transporting the patient directly to a stroke unit without passing through the ED. We also wanted to estimate the delay from the emergency call to admission to a stroke unit. In order further to estimate the relative influence of this strategy on outcome and delay, one control group and one historical reference group were created.

## Methods

Sahlgrenska University Hospital (SU) in Gothenburg, Sweden, is actually three separate hospitals with separate emergency departments and stroke units. Together, they serve a population of approximately 700,000 inhabitants. The present study was performed at one of these three stroke units, SU/Östra, which has 32 in-patient beds and treats approximately 500 stroke patients a year. The patients are treated at the unit for the whole of their hospital stay, both in the acute phase and throughout rehabilitation.

In September 2008, a collaboration project between the EMS system and the hospital was started. The aim of the project was to determine whether selected stroke patients could be identified in the ambulance and be admitted directly to a stroke ward without passing through the ED.

Before the project started, the EMS nurses participated in a 3-hour education course on stroke and stroke symptoms. At the start, the project only involved 2 ambulances, but, in 2009, the project gradually expanded and, by November 2009, most of the 19 ambulances in the area were taking part in the project. In all ambulances, a nurse was on duty at all times. The EMS nurse recorded an ECG, took a blood sample for plasma glucose, measured blood pressure, oxygen saturation (POX), body temperature with an ear-lobe probe and respiratory rate. If plasma glucose was less than 4 mmol/L, the patient was treated with 30% glucose intravenously in the ambulance.

A protocol was used and inclusion and exclusion criteria were registered.

The inclusion criteria were acute onset of a neurological deficit, such as a sudden problem of speaking or a sudden weakness in the face, arm, or leg.

The exclusion criteria were a) patients who met the criteria for i.v. thrombolysis (symptoms for less than 3 hours and under 80 years of age), b) ischemic ST changes on the ECG, c) plasma glucose of 22 mmol/L or more, d) body temperature of 39 degrees Celsius or more, e) POX below 90%, f) systolic blood pressure below 100 mmHg, g) heart rate below 50 or above 100 beats per minute, h) respiratory rate over 25 breaths per minute or i) low consciousness defined as Glasgow Coma Scale of 14 or below. These exclusion criteria were based on the Medical Emergency Triage and Treatment System (METTS) triage system [11].

If a patient met the criteria for direct admission, the EMS nurse contacted a co-ordinator at the stroke unit who double-checked that the inclusion and exclusion criteria had been met and then decided whether or not the patient could be admitted directly to the ward. The co-ordinator was either a nurse or an assistant nurse, so no physician was involved in the decision to admit the patient.

During the study period, direct admission was possible Monday through Friday between 8 am and 4 pm.

All consecutive patients who were admitted directly to the stroke unit between 15 September 2008 and 2 November 2009 were included in the study and formed the direct admission group.

A control group was recruited from consecutive patients who came to the ED by ambulance during the same time period as the direct admission group, on weekdays (Monday through Friday) between 7 am and 6 pm, met the same inclusion and exclusion criteria and where the physician on duty suspected acute stroke and the patient was admitted to the same stroke unit. The patients in the control group all arrived in ambulances not involved in the direct admission project and were therefore not considered for direct admission. The time for inclusion in the control group was extended to 7 am until 6 pm in order to increase the sample size.

Data were collected retrospectively from the patients’ medical charts and ambulance charts.

### Power calculation

An historical reference group was used for the power calculation. It consisted of consecutive patients who came to the ED by ambulance between 1 January and 31 August 2008 on weekdays (Monday through Friday) between 8 am and 4 pm, met the same exclusion criteria according to METTS and were hospitalised with a discharge diagnosis of acute stroke.

This group consisted of 90 patients; 46 men (51%) and 44 women. Their median age was 80 years, Inter quartile range (IQ range): 70–87 years). The median time spent at the emergency ward was 334 minutes (IQ range 245–444 minutes) and the mean delay at the emergency ward was 368 minutes. We estimated the mean time from the onset of the emergency call to arrival at the emergency ward as 40 minutes. The mean time from calling for an ambulance to arrival at the stroke unit was therefore 408 minutes in the historical control group.

Based on experience from fast tracking in acute coronary syndrome (9), we hypothesised that the mean delay from the emergency call to arrival at the stroke unit in the direct admission group would be 60 minutes. If this hypothesis was true, 10 patients in each group would be required (p < 0.05) with 80% power to show a significant shortening of time from the emergency call to arrival at the stroke unit.

### Statistics

All analyses were performed using the SAS© software. Differences in proportions were analysed with Fisher’s exact test. For continuous variables, the Mann–Whitney U test was used.

All tests were two-tailed. P-values of less than 0.05 were considered significant.

### Ethics

According to the criteria set by the Swedish Ethical Review Board, this study was classified as a quality project and evaluation by the Ethical Review Board was therefore not required.

The study was approved by the hospital management committee and informed consent was obtained from all patients included in the project.

## Results

During the study period (September 2008 - November 2009), 53 patients were admitted directly to the stroke unit (direct admission group) and 49 patients met the same inclusion and exclusion criteria at the ED (control group).

### Previous medical history

In Table 1, the two groups are compared in terms of baseline characteristics. The median age was high and the direct admission group was slightly older than the control group (p = 0.048). A higher percentage of patients in the direct admission group had had a prior stroke compared with the control group (51% versus 31%, p = 0.045). In the control group, there were more patients with a diagnosis of ischemic heart disease (35% versus 15%, p = 0.037). There were no other significant differences in terms of distribution of gender, previous medical history, length of stay in hospital or mortality.

**Table 1:** Baseline characteristics and discharge diagnoses for direct admission group (DAG) and control group (CG)

	**DAG (n = 53)**	**CG (n = 49)**	**P**
Age (median, IQ range)	83 (76–88)	79 (68–87)	**0.048**
Gender			0.69
Male	23 (43%)	24 (49%)	
Female	30 (57%)	25 (51%)	
Prior stroke	27 (51%)	15 (31%)	**0.045**
Hypertension	35 (66%)	30 (61%)	0.68
Diabetes mellitus	8 (15%)	6 (12%)	0.78
Atrial fibrillation	13 (25%)	13 (27%)	0.82
Hyperlipidemia	15 (28%)	13 (27%)	1.00
IHD	8 (15%)	17 (35%)	**0.037**
Current smokers (7/3)*	8 (17%)	10 (22%)	0.79
Length of stay in hospital, days	10 (5–19)	13 (7–23)	0.35
(median, IQ range)			
Time EC – ward (1/0)*	54 (45–63)	289 (215–439)	**<0.0001**
(median, IQ range)			
Moved to other ward in hospital	3 (6%)	4 (8%)	0.71
Mortality in hospital	1 (2%)	3 (6%)	0.35
Discharge diagnosis:			0.60
Stroke	29 (55%)	34 (70%)	
TIA	6 (11%)	3 (6%)	
Sequelae of prior stroke	10 (19%)	7 (14%)	
Epilepsy	3 (6%)	1 (2%)	
Other diagnosis	5 (9%)	4 (8%)	

* Number of patients with missing information in each group respectively.

IQ = Inter quartile.

EC = Emergency Call.

Time EC – ward = Time from the patient’s call for ambulance until in patient bed at stroke unit, in minutes.

IHD = Ischemic heart disease.

TIA = Transient Ischemic Attack.

Moved to other ward in hospital = Number of patients who were moved toanother ward in the hospital during hospital stay because of medical condition.

### Delays

The time from calling for an ambulance until arrival at a stroke unit was significantly shorter in the direct admission group than in the control group; median time 54 minutes versus 289 minutes, p < 0.0001.

The mean time between the ambulance being started and arrival at the hospital was 7 minutes longer in the direct admission group compared with the control group (48 minutes versus 41 minutes).

### Diagnostic accuracy

With regard to discharge diagnosis, no significant difference was found between the two groups. Fifty-five per cent of the patients in the direct admission group and 70% in the control group had a final diagnosis of stroke. A final diagnosis of stroke, transient ischemic attack (TIA) or the sequelae of prior stroke was found in 85% and 90% respectively.

The “other diagnosis” group included fainting because of orthostatic hypotension, dizziness, confusion due to urinary tract infection, paresthesia, cerebral tumour and adverse effects of medication.

In the direct admission group, 50 of 53 patients (95%) were treated at the stroke unit during the whole of their hospital stay. Only one of the patients admitted to the stroke unit directly from the ambulance was not in need of hospital treatment as assessed by the physician at the stroke unit. This patient was discharged from hospital after a few hours of observation.

### Complications

In Table 2, patients with a final diagnosis of acute stroke are compared (direct admission group versus control group). Length of stay, various complications, mortality after 3 months and percentage of patients living at home 3 months after stroke did not differ significantly between the groups.

**Table 2:** Comparison of length of stay, complications, mortality and status after 3 months for patients with stroke in direct admission group (DAG) and control group (CG)

	**DAG (n = 29)**	**CG (n = 34)**	**P**
Length of stay in hospital, days	19 (11–24)	16 (9–28)	0.96
(median, IQ range)			
Pneumonia	2 (7%)	1 (3%)	0.59
Venous thromboembolism	0 (0%)	1 (3%)	1.00
Mortality 3 months after stroke	4 (14%)	5 (15%)	1.00
Living at home 3 months			
after stroke (of those living	15/21 (71%)	20 /32 (62%)	0.56
at home before)			

IQ = Inter quartile.

## Discussion

The principle of pre-hospital diagnosis by the EMS system has been introduced for various conditions with the aim of transporting patients directly to special units at hospitals where treatment can be rapidly initiated. This approach has previously been used for patients with acute coronary syndrome, for stroke patients eligible for intravenous thrombolysis and for patients with hip fractures [12,13]. The uniqueness of the present study is that the decision to admit the patient was made entirely by the nurse in the ambulance and a health care provider on the ward using strict criteria. No physician was therefore involved in the decision to admit the patient.

The delay between the first contact with health care and the start of treatment is called system delay. Knowledge of the value of a pre-hospital diagnosis and its possible association with a reduction in system delay is limited. A number of aspects need to be highlighted.

### Diagnostic accuracy

It is well known that many other conditions can imitate acute stroke in the initial phase and previous studies have shown that as many as 30% of patients with an initial suspicion of stroke are found to have other diagnoses [14]. We found that acute stroke was the final diagnosis in 55% of direct admission patients, another 11% were found to have a TIA diagnosis and as many as 19% had a temporary worsening of neurological deficits from a prior stroke, often due to an infection. As a result, 85% of the patients in the direct admission group were found to have a stroke-related diagnosis. The corresponding figure for the control group was 90%. No significant difference between the groups was found with respect to diagnosis at discharge.

With the future development of an appropriate feedback system to the EMS staff, diagnostic accuracy might improve.

The sample size in this pilot study was small and the confidence limits were wide. We need larger studies in order adequately to address the diagnostic accuracy in terms of suspected stroke in the pre-hospital setting.

### System delay

First, we have to acknowledge that system delay is the time from the first contact with health care until the start of treatment, but we looked at the time from the emergency call to arrival at a stroke unit.

Moreover, our findings of a marked reduction in the delay when comparing direct admission with the normal situation, where patients were transported to the ED, should be related to the situation in our community. In other communities, the delay until arrival at a stroke unit might differ both in the direct admission group and in the control group. Our data therefore only illustrate possible achievements with direct admission to a stroke unit when stroke is suspected.

### Impact of direct admission on prognosis

The ultimate goal of direct admission is to reduce the time to delivery of treatment. With the exception of ST-elevation myocardial infarction, there is no evidence that direct admission will improve outcome. For patients with acute stroke, it is essential not only to be diagnosed early but also to start rehabilitation as quickly as possible [2]. Direct admission theoretically offers a better opportunity to achieve this goal.

In this pilot study, we found no significant difference between groups in terms of mortality after 3 months, living at home after 3 months, length of hospital stay or rate of complications. In order to address these questions, a much larger study population would be needed.

### The patients’ perspective

Although difficult to assess, experiencing a stroke is in many cases most probably associated with a high degree of anxiety. It is not unlikely that a shortening of system delay, thereby reducing the time to definite care at the unit where health care providers with experience of stroke treatment take responsibility, would be a positive experience for the patient. Due to the retrospective design of this study, these aspects were impossible to assess. Unfortunately, even in prospective studies, such evaluations will be difficult to perform, as some patients (for example, those with aphasia) will be excluded from the analysis. For this reason, we may still be left in the future with a strong hypothesis that direct admission to a stroke unit, from a patient perspective, is an attractive alternative.

### The ED perspective

One common problem in many hospitals is the large number of patients at the ED [15]. As a result of this, many patients have to wait several hours before being admitted to a ward and they may therefore suffer from adverse reactions. One positive effect of direct admission to a stroke unit is that none of these patients come to the ED, thereby reducing the burden of clinical work in the ED.

On the other hand, if the diagnostic accuracy of the EMS staff’ is low, a negative experience might be that patients without stroke-associated diseases are admitted to stroke units.

### The EMS system perspective

One negative effect of the direct admission routine is that it can be more time consuming for the EMS staff to deliver the patient to the stroke unit rather than to the ED. Previous experience from other direct admission routines shows that the EMS requires an extra 30 minutes [13].

In the present study, the mean time between the ambulance being started and arrival at the hospital was only 7 minutes longer in the direct admission group compared with the control group. However, 7 minutes can be very important if there is an urgent call as a result of cardiac arrest or other life-threatening conditions, for example. Furthermore, we lack information on the prolongation of time until the EMS staff are free for a new mission.

If direct admission becomes a routine for various emergency situations including stroke, myocardial infarction and hip fractures, the burden on the EMS system will most probably increase markedly.

### Selection bias

We used strict criteria for inclusion in the study, the same for both groups, and selected special ambulances for the direct admission group, while other ambulances transported patients to the ED (control group) in order to minimise the risk of selection bias. In spite of this, we are unable to rule out the possibility that selection bias might exist. As many as 51% of patients in the direct admission group had experienced a prior stroke. The fact that an earlier stroke event had occurred might influence the ambulance nurse to find the patient suitable for direct admission. Late neurological deficit after a prior stroke was also found to be a surprisingly common discharge diagnosis (19%) in the direct admission group.

Patients suitable for intravenous thrombolysis were excluded in this study as they have already experienced a fast-track routine. For this reason, we believe that most patients with stroke symptoms are suitable for fast track, irrespective of whether or not they are treated with thrombolysis.

There were three important reasons for the relatively small number of patients in the direct admission group. 1/ Inclusion took place during 40 hours a week (24% of the total week), 2/ at the beginning of the survey, only 2 of 19 ambulances transported patients with presumed stroke directly to a stroke unit and 3/ there were a number of exclusion criteria.

### Future directions

Until the pre-hospital diagnosis of stroke and direct admission to a stroke unit becomes an established principle of treatment, we need to know more. Without doubt, this approach will shorten system delay. However, two critical aspects need to be followed closely. One is the diagnostic accuracy of the EMS staff. In this context, the development of feedback systems to the EMS staff must be introduced in order to improve their diagnostic accuracy even further. Furthermore, the extra burden on the EMS system must be further clarified, with the aim of optimising the balance between resources and performance within the EMS system.

## Conclusions

In a pilot study, the concept of a pre-hospital diagnosis of stroke by a nurse was associated with relatively high diagnostic accuracy in terms of stroke-related diagnoses and a short delay to arrival at a stroke unit. These data need to be confirmed in larger studies, with a concomitant evaluation of the clinical consequences and, if possible, patient satisfaction.

## Competing interests

The authors declare that they have no competing interests.

## Authors’ contributions

IW, PK, JH, MK and PH designed the study. BL, CG and PH included the patients. All authors participated in the draft of the manuscript. All authors read and approved the final manuscript.
